# Berberine Promotes Glucose Consumption Independently of AMP-Activated Protein Kinase Activation

**DOI:** 10.1371/journal.pone.0103702

**Published:** 2014-07-29

**Authors:** Miao Xu, Yuanyuan Xiao, Jun Yin, Wolin Hou, Xueying Yu, Li Shen, Fang Liu, Li Wei, Weiping Jia

**Affiliations:** 1 Shanghai Clinical Center for Diabetes, Shanghai Clinical Center for Metabolic Diseases, Shanghai Key Laboratory of Diabetes Mellitus, Shanghai Diabetes Institute, Department of Endocrinology and Metabolism, Shanghai Jiao Tong University Affiliated Sixth People’s Hospital, Shanghai, China; 2 Department of Clinical Nutrition, Shanghai Jiao Tong University Affiliated Sixth People’s Hospital, Shanghai, China; Northwestern University, United States of America

## Abstract

Berberine is a plant alkaloid with anti-diabetic action. Activation of AMP-activated protein kinase (AMPK) pathway has been proposed as mechanism for berberine’s action. This study aimed to examine whether AMPK activation was necessary for berberine’s glucose-lowering effect. We found that in HepG2 hepatocytes and C2C12 myotubes, berberine significantly increased glucose consumption and lactate release in a dose-dependent manner. AMPK and acetyl coenzyme A synthetase (ACC) phosphorylation were stimulated by 20 µmol/L berberine. Nevertheless, berberine was still effective on stimulating glucose utilization and lactate production, when the AMPK activation was blocked by (1) inhibition of AMPK activity by Compound C, (2) suppression of AMPKα expression by siRNA, and (3) blockade of AMPK pathway by adenoviruses containing dominant-negative forms of AMPKα1/α2. To test the effect of berberine on oxygen consumption, extracellular flux analysis was performed in Seahorse XF24 analyzer. The activity of respiratory chain complex I was almost fully blocked in C2C12 myotubes by berberine. Metformin, as a positive control, showed similar effects as berberine. These results suggest that berberine and metformin promote glucose metabolism by stimulating glycolysis, which probably results from inhibition of mitochondrial respiratory chain complex I, independent of AMPK activation.

## Introduction

Berberine, a plant isoquinoline alkaloid, is used as an over-the-counter drug in China to treat infectious diarrhea. The Chinese name of berberine is Huangliansu, which means the element of Rhizoma Coptidis. Rhizoma Coptidis is a popular traditional Chinese herb used for treatment of inflammation and diabetes, and contains 5.2%–7.7% of berberine. The anti-diabetes activity of Rhizoma Coptidis is documented as early as 1500 years ago. However, Rhizoma Coptidis was usually used to treat infection or inflammation since diabetes was not popular in ancient time [1], [2].

The beneficial effects of berberine on metabolism are pleiotropic. In folk medicine of China, berberine is used to treat diabetes, obesity and non-alcoholic fatty liver disease [3]–[7]. In addition, berberine is also reported to alleviate dyslipidemia and cardiovascular diseases [8]–[11]. A variety of molecular mechanisms were proposed for the anti-diabetic effect of berberine, such as AMPK activation [12], [13], inhibition of PPARγ and C/EBPα function [14], [15], antioxidant [16], inhibition of aldose reductase and inhibitory activities on MAPK [1], [17]–[19]. Among them, AMPK activation is considered as the major mechanism. AMPK is a highly conserved sensor of cellular energy status that exists in almost all eukaryotes; phosphorylation of Thr-172 is used as a biomarker of AMPK activation, which can be enhanced by berberine and metformin. Once activated, AMPK promotes catabolic processes (glycolysis, fatty acid oxidation, etc.), while turning off anabolic pathway (glycogen, cholesterol and protein synthesis, etc.) [20]. Thus, AMPK pathway is one of the most important drug targets for metabolism regulation.

However, our previous study indicated that berberine enhanced glucose metabolism via stimulation of glycolysis, which was related to inhibition of glucose oxidation in mitochondria [21]. Since stimulating glycolysis could be a direct consequence of mitochondrial inhibition, we hypothesize that berberine’s effect on glucose metabolism is related to mitochondrial inhibition, independent of AMPK activation. In this context, we evaluated underlying mechanisms of berberine’s effects on glycolysis and glucose consumption with emphasis on the roles of AMPK and mitochondrial respiratory chain complex I. Metformin, another well-known AMPK activator, was used as a positive control to verify the activation of AMPK in our cellular models. Our results suggest both berberine and metformin promote glucose consumption independently of AMPK activation.

## Materials and Methods

### Reagents

Berberine was obtained from the National Institute for the Control of Pharmaceutical and Biological Products (Beijing, China). Metformin was purchased from Shanghai Sangon Biotechnology Corporation (Shanghai, China). Both drugs were dissolved in ddH2O, which was used as the vehicle for berberine and metformin. Compound C was purchased from Merck KGaA (Darmstadt, Germany) and dissolved in DMSO, which was used as the vehicle for compound C. Dulbecco’s modified Eagle’s medium (DMEM), fetal bovine serum (FBS), and other culture reagents were purchased from Gibco Life Technologies (Grand Island, NY). Rotenone, sodium pyruvate, GlutaMax-1 and other chemical reagents were purchased from Sigma Chemicals (St Louis, MO).

### Cells

The human hepatoma cell line HepG2 and mouse skeletal myoblast C2C12 were maintained in a 37°C, 5% CO_2_ incubator and cultured in a growth medium: DMEM supplemented with 10% fetal bovine serum, 100 units/ml penicillin and 0.1 mg/ml streptomycin (low-glucose DMEM for HepG2 cells, and high-glucose DMEM for C2C12 cells). For differentiation of myotubes, C2C12 myoblasts were seeded into 12-well plates in DMEM with 10% FBS for 24 h. Then the medium was replaced by differentiation medium: DMEM containing 2% horse serum, 100 units/ml penicillin and 0.1 mg/ml streptomycin for 6 days. The medium was refreshed every 48 h.

### Glucose consumption

The cells were cultured in 96-well plates and treated with berberine or metformin at various concentrations in FBS-free DMEM (15 mmol/L D-glucose) supplemented with 0.25% BSA for 24 h. The glucose concentration in the medium was determined by the glucose oxidase method. The amount of glucose consumption was calculated by subtracting the glucose concentration of cells treated with berberine or metformine from the cells treated with vehicle [22], [23].

### LDH cytotoxicity assay

The percentage of living cells was determined using the Lactate Dehydragenase (LDH) Cytotoxicity Assay Kit (Beyotime Institute of Biotechnology, Jiangsu, China). In brief, HepG2 hepatocytes and C2C12 myotubes were cultured in 96-well plates. After berberine or metformin treatment for 24 h in DMEM supplemented with 0.25% BSA, LDH was measured in medium and cell extracts according to the manufacturer’s instruction.

### RNA interference

One day before transfection, HepG2 cells were placed into 24-well plates. When cell densities reached 50% confluent, they were transfected with siRNA directed against AMPKα1/α2 (sc-45312; Santa Cruz, CA, USA) or scrambled siRNA (sc-37007; Santa Cruz, CA, USA) as a control. Briefly, cells were transfected with 30 pmol/well siRNA via Lipofectamine 2000 Transfection Reagent (11668; Invitrogen, Carlsbad, CA, USA) according to the manufacturer’s instructions.

### Adenovirus Infection

Recombinant adenoviruses expressing dominant negative forms of AMPK, AMPKα1 (D159A) and AMPKα2 (K45R) (α1/α2-DN), were gifts from Dr. Jia Li at Shanghai Institute of Materia Medica, Chinese Academy of Sciences. The α1/α2-DN were used to block AMPK activity as previously described [24], [25]. In brief, HepG2 cells were infected with adenovirus expressing control GFP reporter protein or dominant site mutagenesis AMPKα1/α2, hereinafter referred to as DN-AMPK, for 5–6 h and treated with berberine or metformin for 24h after infection.

### Western blot

Cells were washed with ice-cold phosphate-buffered saline (PBS) and lysed with lysis buffer (50 mM Tris-HCl, 150 mM NaCl, 1% Triton X-100, 1% sodium deoxycholate, 0.1% SDS, 1 mM sodium orthovanadate, 1 mM sodium fluoride, 1 mM EDTA, 10 µg/ml leupeptin, 1 mM PMSF, and phosphatase inhibitor cocktail; pH 7.4). The extracted protein (35 µg) was boiled for 5 min, subjected to SDS-polyacrylamide gel electrophoresis (SDS-PAGE). Then the separated proteins were transferred onto a nitrocellulose membrane. After blocked with 5% skim milk in the TBST buffer for 1 h, the membrane was incubated with primary antibody at 4°C overnight. Antibodies to AMPK, acetyl coenzyme A synthetase (ACC), phospho-AMPK (Thr172), β-actin and phospho-ACC (Ser-79) were purchased from Cell Signaling Technology (Danvers, MA). The HRP-conjugated secondary antibodies (Promega Corporation, Madison, USA) were used with chemiluminescence reagent (Thermo Fisher Scientific Inc., Rockford, USA) for generation of the light signal; Gel-Pro Analyzer 4.0 was used to quantify the Western signals.

### Determination of lactate content

The cells were cultured in 96-well plates and treated with berberine or metformin in DMEM supplemented with 0.25% BSA for 24 h. The lactate concentration in the medium was measured with a lactate reagent kit (Shanghai Juchuang Biotechnology Corporation, Shanghai, China).

### Extracellular flux (XF) analysis

C2C12 myoblasts were seeded in XF 24-well cell culture microplates (Seahorse biosciences, North Billerica, MA) at 1.25×10^4^ cells/well in 250 µl of growth medium and placed in a 37°C incubator with 5% CO_2_. Once the cells achieved confluence, differentiation medium was used to induce the myotube differentiation. The medium was refreshed every other day. After 6 days, C2C12 myotubes were treated with 20 µmol/L berberine or 5 mmol/L metformin for 24 h, followed by XF bioenergetic assay. Assays were initiated by replacing the medium with 675 µl of assay medium (8.3 g/l DMEM Base, 1.85 g/l NaCl, 2 mM GlutaMax-1, 1mM sodium pyruvate, 15 mM D-glucose, 20 mM HEPES and 15 mg of Phenol Red; pH 7.4) prewarmed to 37°C. Cells were incubated at 37°C without CO_2_ for 60 min to allow cells to preequilibrate with the assay medium before the first measurement.

After the equilibration period, cells were subjected to three baseline measurements, followed by injection of the following reagents: 1 µM oligomycin, an inhibitor of ATP synthesis was used to distinguish O_2_ consumption devoted to ATP synthesis; 1 µM FCCP (Carbonyl cyanide-p-trifluoromethoxyphenylhydrazone), an uncoupling agent was added to measure uncoupled respiration; 1 µM rotenone, a complex I inhibitor was used to assay complex I-linked respiration [26], [27].

### Statistical analysis

Data are presented as means ± SEM form individual experiments. All experiment were performed at least in triplicate. Student’s *t*-test or one-way ANOVA (SPSS 17.0) was used in statistical analysis of the data, with *P<*0.05 considered significant.

## Results

### Berberine inhibited respiratory chain complex I and reduced ATP synthesis in C2C12 myotubes

**Figure 1:**
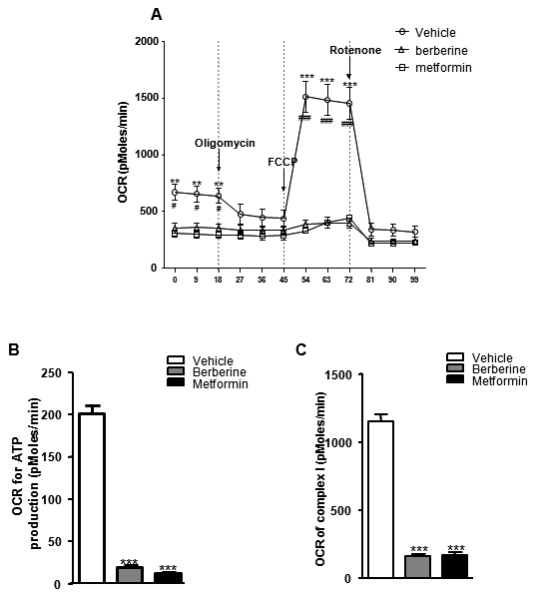
Effects of berberine on ATP synthesis and complex I - linked respiration in C2C12 myotubes. Cells were treated with berberine (20 µmol/L) or metformin (5 mmol/L) for 24 h, followed by XF bioenergetic assay. A: Traces of OCRs in control, berberine- or metformin-treated C2C12 cells followed the addition of oligomycin, FCCP and rotenone. Results are shown as means ± SEM, *n = *3.^ #^ *P<*0.05, ^###^ *P<*0.001 *vs*. berberine-treated cells; **P<0.01, ***P<0.001 *vs*. metformin-treated cells in corresponding group. B: Effects of berberine and metformin on OCR for ATP production. C: Effects of berberine and metformin on complex I - linked oxygen consumption. Results are shown as means ± SEM, *n = *3; ***P<0.001 *vs*. control in corresponding group.

Extracellular flux analysis was performed to assess the mitochondrial respiration in C2C12 myotubes. As shown in Fig. 1A, treatment of berberine or metformin for 24 hours significantly decreased the basal oxygen consumption rates (OCR). The decrease in OCR resulted from blocking ATP synthetase by oligomycin was used as a marker of ATP synthesis. Compared with control, berberine or metformin significantly reduced ATP synthesis in C2C12 myotubes (Fig. 1B). FCCP, a chemical uncoupler of electron transport and oxidative phosphorylation, increased OCR to the maximum. Rotenone inhibited respiratory chain complex I and reduced the OCR to the minimum. The difference in ORC induced by these two compounds was the O_2_ consumption incurred by complex I, and used to evaluate complex I - linked respiration. Complex I - linked respiration was almost abolished with the treatment of berberine or metformin (Fig. 1C). Nevertheless, there was no significant alteration in complex II - linked respiration between control and treatment groups (Fig. S1).

### Berberine had no cytotoxicity

**Figure 2:**
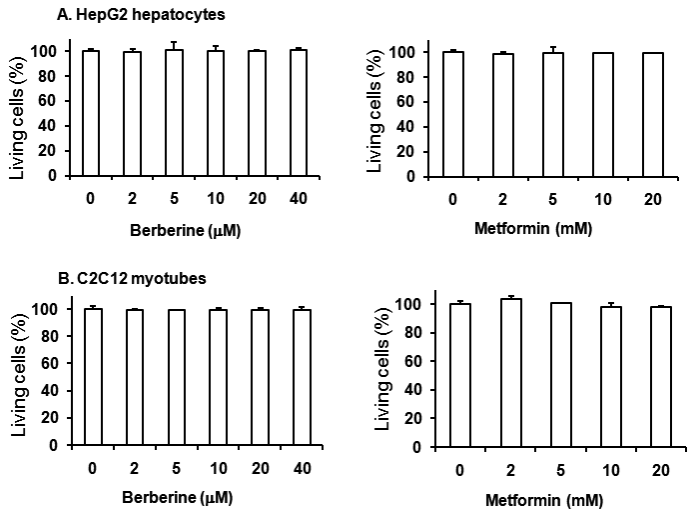
Berberine showed no cytotoxicity. Cells were starved in serum-free DMEM supplemented with 0.25% BSA and treated with various doses of berberine or metformin for 24 h. LDH concentrations in medium and cells were detected. A: berberine or metformin treatment had no cytotoxicity in HepG2 hepatocytes. B: berberine or metformin treatment had no cytotoxicity in C2C12 myotubes. Data are expressed as means ± SEM; *n = *8.

The data above suggested that berberine or metformin inhibited respiratory chain complex I. It was necessary to know whether these drugs had toxicity in the cells. Thus, LDH cytotoxicity assay was conducted to address this issue. Obviously, in both HepG2 hepatocytes and C2C12 myotubes, neither berberine nor metformin increased LDH release during the exposure duration up to 24 h (Fig. 2). These data suggested that berberine or metformin did not exhibit toxicity in our experimental system.

### Berberine increased lactate release *in vitro*

**Figure 3:**
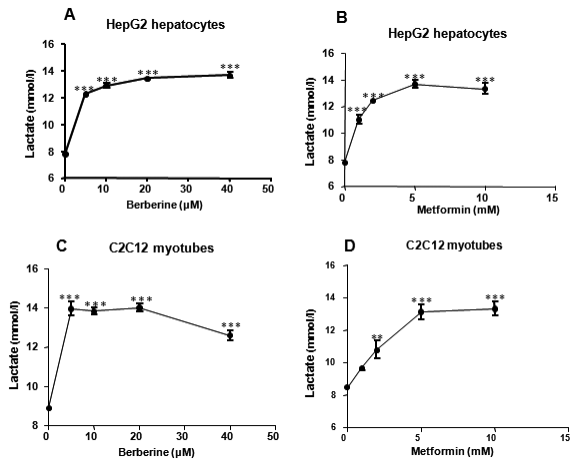
Berberine increased lactate release in a dose-dependent manner in cultured cells. Cells were cultured in 96-well plates. Lactate concentration was measured after treatment with increasing concentrations of berberine or metformin in serum-free medium for 24 h. A: Effect of berberine on lactate release in HepG2 cells. B: Effect of metformin on lactate release in HepG2 cells. C: Effect of berberine on lactate release in C2C12 cells. D: Effect of metformin on lactate release in C2C12 cells. Data are expressed as means ± SEM; *n = *4. **P<0.01 and ***P<0.001 *vs*. corresponding control (0 mol/L).

To investigate the effect of berberine on anaerobic respiration, lactate release was measured in HepG2 hepatocytes and C2C12 myotubes. HepG2 cells were incubated for 24 h in the presence or absence of 5, 10, 20 or 40 µmol/L berberine. Lactate release of HepG2 hepatocytes was increased by berberine in a dose-dependent manner. The concentration of lactate increased by 57.25% to 75.13% (*P<*0.001; Fig. 3A). Incubation with 1 to 10 mmol/L metformin for 24h, lactate release of HepG2 hepatocytes was increased by 41.0% to 74.8% (*P<*0.001; Fig. 3B). Similar effect was observed in C2C12 myotubes (*P<*0.01 to *P<*0.001; Fig. 3C and D). Lactate is the end product of glycolysis. These data suggested that berberine may induce anaerobic respiration in vitro.

### Berberine improved glucose consumption *in vitro*

**Figure 4:**
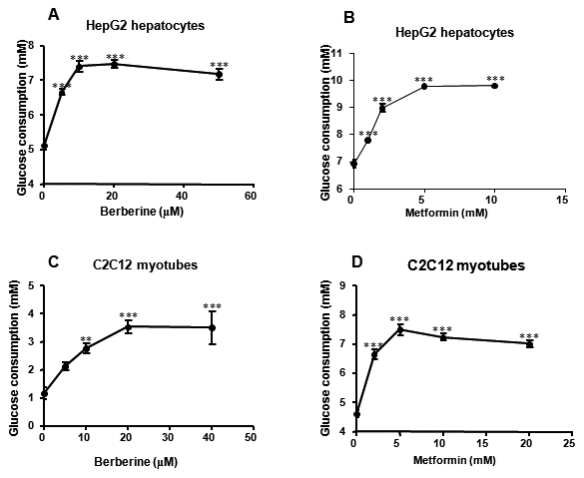
Berberine promoted glucose consumption in a dose-dependent manner in HepG2 hepatocytes and C2C12 myotubes. Cells were cultured in 96-well plates firstly. During experiments, culture medium was replaced by serum-free DMEM supplemented with 0.25% BSA and 15 mM D-glucose. Glucose consumptions were measured after 24-hour incubation with increasing concentrations of berberine or metformin. A: Effect of berberine on glucose consumption in HepG2 cells. B: Effect of metformin on glucose consumption in HepG2 cells. C: Effect of berberine on glucose consumption in C2C12 cells. D: Effect of metformin on glucose consumption in C2C12 cells. Data are expressed as means ± SEM from at least four independent experiments. **P<0.01 and ***P<0.001 *vs*. corresponding control (0 mol/L).

The data above suggested that berberine affected cellular respiration by inhibiting aerobic respiration and stimulating anaerobic respiration. It was known that glycometabolism was closely related to cellular respiration. To identify the effect of berberine or metformin on glycometabolism, glucose consumption was examined in HepG2 hepatocytes and C2C12 myotubes after 24 h treatment of berberine or metformin. In HepG2 hepatocytes, at concentrations between 5 and 20 µmol/L, berberine increased glucose consumption by 29.91% to 45.91% (*P<*0.001; Fig. 4A). Metformin increased glucose consumption in a dose-dependent manner. In doses of 1, 2, 5 and 10 mmol/L, the glucose consumption was increased by 12.59% to 41.74% (*P<*0.001; Fig. 4B). Similar effects of these two drugs were observed in C2C12 myotubes (Fig. 4C and D).

### Berberine induced AMPK phosphorylation in HepG2 hepatocytes and C2C12 myotubes

**Figure 5:**
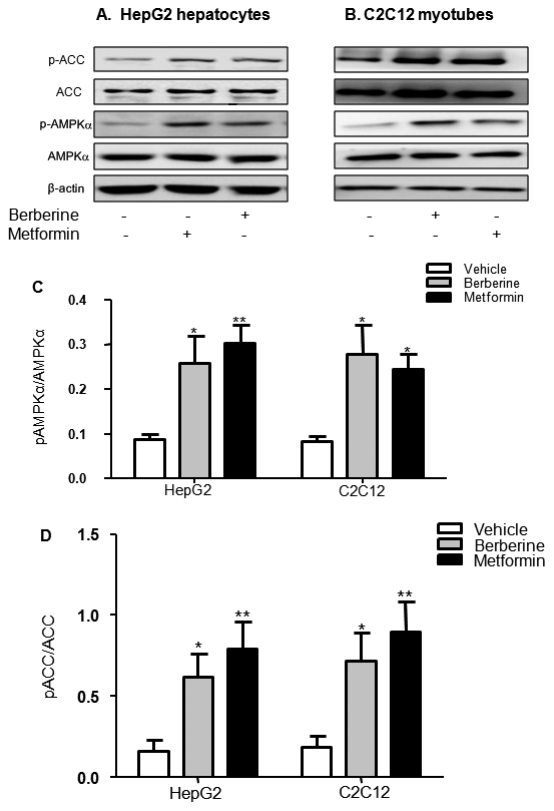
Effects of berberine on phosphorylation of AMPK and ACC. HepG2 hepatocytes (A) and C2C12 myotubes (B) were treated with 20 µmol/L berberine or 10 mmol/L metformin for 24 h after serum starvation in DMEM with 0.25% BSA and 15 mM D-glucose. Western blot was performed with the whole cell lysate. Stimulation of AMPK and ACC phosphorylation was quantified as relative optical density (C and D). Results are shown as means ± SEM from at least six independent experiments; *P<0.05 and **P<0.01 *vs*. control.

Effects of berberine on AMPK activity were detected in HepG2 hepatocytes and C2C12 myotubes. Significant increases of AMPK and ACC phosphorylation were observed after treatment with 20 µmol/L berberine or 10 mmol/L metformin for 24 h. Berberine increased phosphorylation level of AMPK (Thr^172^) and ACC (Ser^79^) by 2.0- and 2.8-fold greater than control in hepatocytes, by 2.4- and 2.8-fold higher than control in myotubes (*P<*0.05), respectively. Meanwhile, metformin resulted in a 2.5- and 3.9-fold increase in hepatocytes, 2.0- and 3.8-fold enhancement in myotubes, respectively (Fig. 5 C and D).

### Berberine-stimulated glucose consumption and lactate release were not blocked by compound C

**Figure 6:**
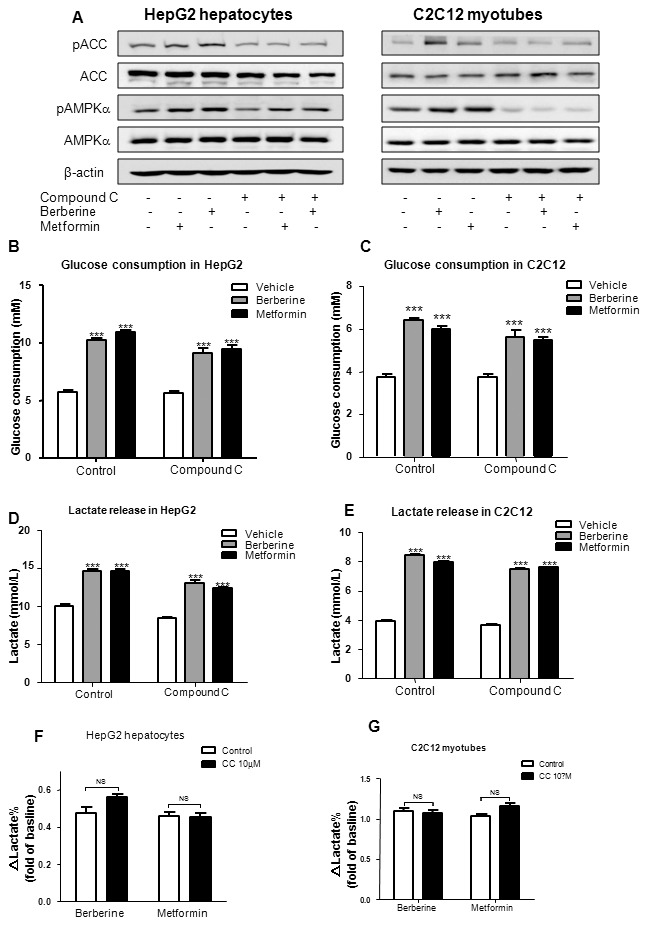
Effects of berberine on AMPK pathway, glucose consumption and lactate release in the presence of compound C. Cells were preincubated with compound C (10 µmol/L) for 30 min, then treated with 20 µmol/L berberine or 10 mmol/L metformin in serum-free DMEM with 0.25% BSA and 15 mM D-glucose for 24 h. Whole cell lysate protein was used to measure the phosphorylation of AMPK (pAMPK) and ACC (pACC) by Western blot in HepG2 hepatocytes and C2C12 myotubes (A). Effects of berberine and metformin on glucose consumption were checked in HepG2 (B) and C2C12 (C) cells; effects of berberine and metformin on lactate release were checked in HepG2 (D) and C2C12 (E) cells. Increasing rates of lactate release stimulated by berberine and metformin in the presence or absence of compound C in HepG2 (F) and C2C12 (G) cells were calculated. Results are shown as means ± SEM from at least three independent experiments; ***P<0.001 *vs*. control in corresponding group.

To determine whether berberine-stimulated glucose consumption and lactate release involved activation of AMPK pathway, the effect of compound C, an AMPK inhibitor, on berberine-stimulated glucose consumption and lactate release were measured. HepG2 hepatocytes and C2C12 myotubes were incubated for 30 min with 10 µmol/L compound C, following treatment with berberine or metformin for 24 h. As shown in Fig. 6A, compound C fully blocked berberine or metformin-induced ACC phosphorylation, and decreased phosphorylation level of AMPK, suggesting it had inhibited AMPK activity in HepG2 hepatocytes and C2C12 myotubes. With treatment of compound C, berberine and metformin still increased glucose consumption by 78.6% and 91.3% (*P<*0.001, *P<*0.001) in HepG2 cells (Fig. 6B), 71.6% and 60.3% (*P<*0.001, *P<*0.001) in C2C12 cells, respectively (Fig. 6C).

As shown in Fig. 6D and E, berberine or metformin significantly increased lactate release in both HepG2 and C2C12 cells (*P<*0.001). In the presence of compound C, the lactate concentration showed a trend of decrease. However, the increasing rates of lactate release stimulated by berberine and metformin had no significant differences between control and compound C groups in both cell lines (Fig. 6F and G).

### Berberine-stimulated glucose consumption and lactate release were not reduced upon AMPKα1/α2 silencing in HepG2 hepatocytes

**Figure 7:**
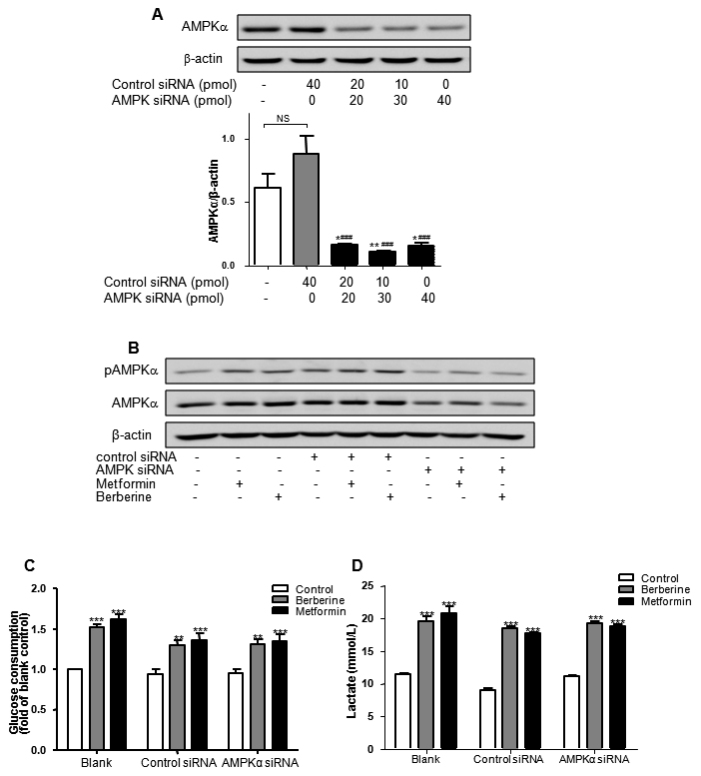
Effects of berberine on glucose consumption and lactate release upon AMPKα1/α2 silencing in HepG2 hepatocytes. A: HepG2 hepatocytes were seeded in 24-well plates, and transfected with various doses of scrambled siRNA and/or AMPKα1/α2 siRNA for 24 h. The protein level was examined by Western blot. Ratio of AMPKα to β-actin was quantified in 3 independent experiments per condition. It had been found that AMPKα1/α2 siRNA at 30 pmol/well most effectively suppressed the expression of AMPKα. *P<0.05 and **P<0.01 *vs*. blank control; ^###^ *P<*0.001 *vs*. control siRNA. B, C and D: After transfection for 24 h, HepG2 hepatocytes were treated with berberine (20 µmol/L) or metformin (10 mmol/L) for another 24 h, followed by Western blot, glucose consumption and lactate concentration assay. Results are shown as means ± SEM from at least three independent experiments; **P<0.01, ***P<0.001 *vs*. control in corresponding group.

To further prove the effect of AMPK on glucose-lowering action of berberine, AMPKα1/α2 siRNA was used to inhibit the expression and activation of AMPKα in HepG2 hepatocytes. As shown in Fig. 7A and B, compared with scrambled siRNA, AMPKα1/α2 siRNA significantly decreased the expression of AMPKα (*P<*0.001), meanwhile berberine or metformin-induced AMPKα phosphorylation was also blocked. It was similar to compound C study. Compared with control, berberine or metformin significantly increased glucose consumption and lactate release in all three groups. Although the levels of glucose consumption and lactate concentration in siRNA transfected HepG2 hepatocytes were slightly below the blank control, there were no significant differences in berberine or metformin induced glucose consumption and lactate release between scrambled siRNA and AMPKα1/α2 siRNA transfection (Fig. 7C and D). The data indicated that AMPKα1/α2 silencing did not reduce the glucose-lowering effect of berberine.

### Berberine-stimulated glucose consumption and lactate release were not reversed in DN-AMPK adenoviruses infected HepG2 hepatocytes

**Figure 8:**
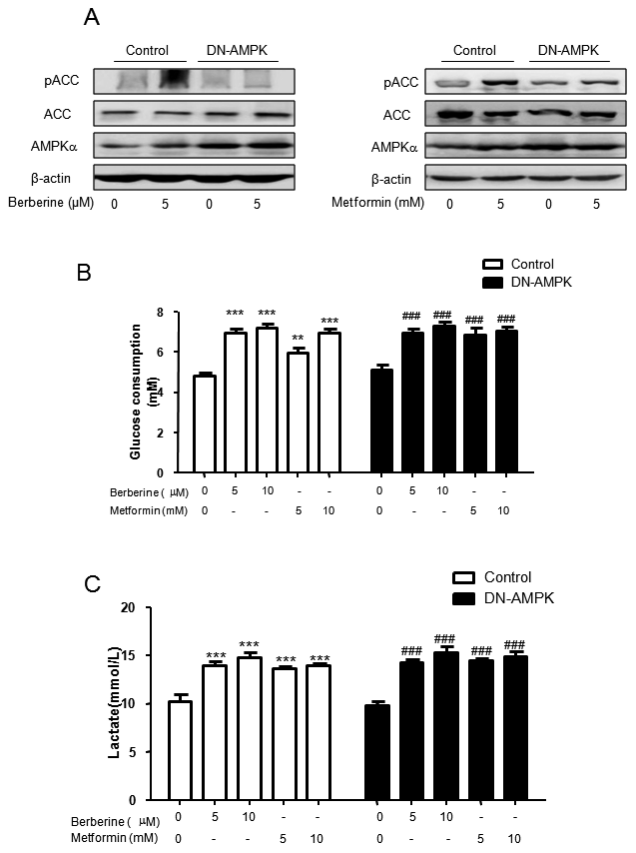
Effects of berberine on glucose consumption and lactate release in DN-AMPK adenovirus infected HepG2 hepatocytes. HepG2 cells were infected with adenoviruses expressing control GFP reporter protein or DN-AMPK for 5–6 h then treated with berberine or metformin for 24h. The protein level was examined using Western blot (A). It had been found that DN-AMPK adenovirus expression reversed berberine- or metformin-induced ACC phosphorylation. However, the enhancement of glucose consumption (B) and lactate release (C) stimulated by berberine and metformin was not altered. Results are shown as means ± SEM from at least three independent experiments; **P<0.01, ***P<0.001, ^###^ *P<*0.001 *vs*. untreated control in corresponding group.

To confirm the hypothesis that hypoglycemic effect of berberine was AMPK-independent, the DN-AMPK adenovirus was employed to completely inhibit AMPK pathway. As shown in Fig. 8A, compared with control GFP reporter protein adenovirus, DN-AMPK adenovirus expressed more AMPKαin hepatocytes, while berberine or metformin-induced ACC phosphorylation was completely reversed. However, the enhancement of glucose consumption and lactate release stimulated by berberine and metformin was not altered (Fig. 8B and C). These data indicate that AMPK pathway is not involved in altered glucose consumption by berberine or metformin.

## Discussion

In the late 1990s, as a result of screening novel hypoglycemic agents from active components of traditional Chinese herbs, we found berberine, a main component of an ancient Chinese herb *Coptis chinensis French.*, had a significant glucose-lowering effect in HepG2 cells [28], [29]. We further compared berberine with other oral hypoglycemic agents and found that the effect of berberine was independent of insulin, which was quite different from that of troglitazone, the first thiazolidinedione used in clinical setting [22], [30]. Based on these findings, we conducted one of the first clinical trials to investigate the efficacy of berberine on diabetes. In newly diagnosed type 2 diabetic patients, hemoglobin A1c was decreased from ∼9.5% to ∼7.5% after berberine treatment for 3 months. The study indicated that berberine was a potent oral hypoglycemic agent with beneficial effects on lipid metabolism [4].

Our current study is to identify whether AMPK is necessary for glucose consumption and glycolysis induced by berberine or metformin. Although Jeong et al. found that the effects of berberine on proinflammatory responses in macrophages were AMPK dependent [32], there is no reported study to investigate if AMPK was essential for the glucose-lowering effect of berberine. In current study, inactivation of AMPK via siRNA, compound C, or DN-AMPK adenovirus, was employed in HepG2 hepatocytes or C2C12 myotubes. We found that in the absence of AMPK activation, berberine still exhibited potent glucose-lowering and glycolysis-inducing effects on human hepatoma cells and mouse myotubes. For the first time, we demonstrated that berberine may promote glucose metabolism independently of AMPK pathway.

A parallel increase in glucose consumption and lactate release was observed in this study. The lactate in the culture medium is derived from glycolysis process. Glycolysis transfers energy from glucose to ATP through an oxygen-independent path outside mitochondria. Compared with aerobic respiration, glycolysis is less efficient in ATP synthesis and requires more glucose to produce the same amount of ATP [21]. Thus, glycolysis enhancement may be the primary mechanism by which berberine increase glucose consumption in hepatocytes and myotubes. Therefore, mitochondrial function was investigated to explore the role of respiratory modification in mediating the glucose-lowering effect of berberine.

The results from the current study indicate that berberine almost completely blocked electron transport train complex I activity. Our previous study revealed that berberine was able to repress mitochondrial function in vitro [21]. Turner et al reported berberine suppressed respiration via complex I in isolated mitochondria [33]. However, it’s important to differentiate pharmaceutical effect from toxic effect of berberine in mitochondria. Thus, intact living cells cultured in a 24-well microplate and the best effective dosage of berberine (20 µmol/L) was chosen in the experiment. Extracellular flux analysis was performed to assess the mitochondrial respiration by Seahorse XF24 analyzer [34], [35]. Complex I - linked respiration was nearly totally blocked by berberine. This is the first study in which respiratory chain complex I inhibition by berberine was observed in living cells. Unexpectedly, the cells grow normally and consuming much more glucose in the absence of complex I activity. The data challenged our concept about complex I, inhibition of which may be a key to solve the disturbance of glucose metabolism.

Our results confirmed that berberine was able to inhibit ATP synthesis in C2C12 myotubes. In extracellular flux analysis, oligomycin reduced OCR, which represents the decreased ATP synthesis [26]. The reduction was nearly erased by berberine. The inhibition of ATP synthesis leads to compensatory enhancement of anaerobic respiration. The decrease of ATP production may elevate AMP/ATP ratio in cells, further leads to the activation of AMPK [36]. Therefore, mitochondrial inhibition might be the initial driving force for glucose-lowering action of berberine.

This study suggests that berberine and metformin may share similar effects and mechanisms in regulation of glucose metabolism. Our previous study showed the hypoglycemic efficacy of berberine was comparable to that of metformin in a clinical trial [4]. However, these two medications have never been compared in ex vivo experiments. This study explored underlying mechanism and pattern of glucose-lowering effect of berberine and metformin in cells. Except effective dose range (1∼10 mmol/L for metformin and 5∼40 µmol/L for berberine), berberine and metformin showed almost identical effects on stimulating glucose consumption and lactate release. For the first time, this study showed that these two medications had similar capacity to activate AMPK pathway and inhibit complex I activity. This indicates they may belong to the same class of hypoglycemic agents despite of totally different chemical structure.

This study also suggested that AMPK pathway may not play a central role in the action of metformin. Up to now, AMPK is still considered as the main target of metformin [37], [38], although Foretz et al found that metformin was able to inhibit gluconeogenesis in AMPKα1α2-null mouse hepatocytes [39]. A recent paper reported that metformin blocked glucagon-dependent glucose output from hepatocytes by decreasing production of cyclic AMP [31]. Some scientists argued that results from mouse genetic model were not always applicable to human body since inactivation of critical enzymes sometimes led to adaptations in alternative pathways [40]. Thus, new strategy was implemented in this study. For the first time, effects of metformin on glucose consumption and glycolysis were studied in cell models of AMPK transient inactivation. Temporary blockade of AMPK failed to diminish the pharmaceutical effects of metformin, too. This study provides evidence that, in addition to inhibition of glucose production, AMPK may also be dispensable for metformin-induced glucose consumption and glycolysis in hepatocytes and myotubes.

In conclusion, the results from the current study demonstrated that berberine inhibited mitochondrial respiratory chain complex I, which led to the suppression of ATP synthesis, and the enhancement of glycolysis. The elevated glycolysis may be a primary cause for increased glucose consumption in hepatocytes and myotubes by berberine. Furthermore, all these effects are independent of AMPK activation. Berberine and metformin showed identical effects in vitro. We suggest complex I inhibition may replace AMPK activation as a major molecular mechanism of berberine and metformin.

## Supporting Information

Figure S1 **Berberine had no effects on complex II - linked respiration in C2C12 myotubes.** To further understand the modulation of mitochondrial respiration by berberine, complex II - linked respiration was also examined by extracellular flux assay in C2C12 myotubes. After complex I - linked respiration was inhibited by rotenone, succinate was further administrated as the substrate for complex II. Then antimycin A was added to inhibit the function of complex III, which is the downstream of complex I and II. The change of OCR was recorded in real time. Since complex I had been already blocked, OCR reduction caused by antimycin A reflected complex II - linked respiration. As shown in Fig. S1, there was no significant change in complex II - linked respiration with the treatment of berberine or metformin. The results suggest that berberine and metformin had no effects on complex II activity. A: Traces of OCRs in control, berberine- or metformin-treated C2C12 cells followed the addition of rotenone, succinate and Antimycin A. B: Effects of berberine and metformin on complex II - linked oxygen consumption. OCRs are expressed as fold of baseline OCRs, and shown as means ± SEM, *n* = 3.
(TIF)
