# Supplementary figures and images for: Berberine Promotes Glucose Consumption Independently of AMP-Activated Protein Kinase Activation
Source: PLoS One. 2014 Jul 29;9(7):e103702. doi: 10.1371/journal.pone.0103702 (PMC4114874; doi:10.1371/journal.pone.0103702)

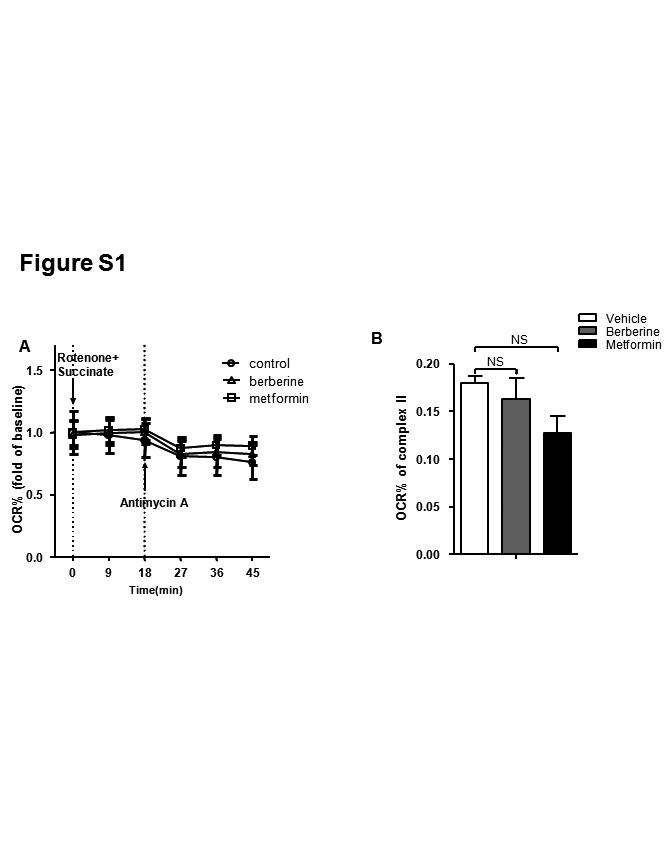

Supplement: Figure S1 — Berberine had no effects on complex II - linked respiration in C2C12 myotubes. To further understand the modulation of mitochondrial respiration by berberine, complex II - linked respiration was also examined by extracellular flux assay in C2C12 myotubes. After complex I - linked respiration was inhibited by rotenone, succinate was further administrated as the substrate for complex II. Then antimycin A was added to inhibit the function of complex III, which is the downstream of complex I and II. The change of OCR was recorded in real time. Since complex I had been already blocked, OCR reduction caused by antimycin A reflected complex II - linked respiration. As shown in Fig. S1, there was no significant change in complex II - linked respiration with the treatment of berberine or metformin. The results suggest that berberine and metformin had no effects on complex II activity. A: Traces of OCRs in control, berberine- or metformin-treated C2C12 cells followed the addition of rotenone, succinate and Antimycin A. B: Effects of berberine and metformin on complex II - linked oxygen consumption. OCRs are expressed as fold of baseline OCRs, and shown as means ± SEM, n = 3. (TIF) [file pone.0103702.s001.tif]
